# SARS-CoV-2 S Protein Reduces Cytoprotective Defenses and Promotes Human Endothelial Cell Senescence

**DOI:** 10.14336/AD.2024.0405

**Published:** 2025-06-25

**Authors:** Alicia Villacampa, Licia Shamoon, Inés Valencia, Cristina Morales, Sofía Figueiras, Fernando de la Cuesta, Dolores Sánchez-Niño, Guillermo Díaz-Araya, Isabel Sánchez-Pérez, Óscar Lorenzo, Carlos Félix Sánchez-Ferrer, Concepción Peiró

**Affiliations:** ^1^Department of Pharmacology, School of Medicine, Universidad Autónoma de Madrid, Spain.; ^2^Vascular Pharmacology and Metabolism (FARMAVASM) group, IdiPAZ, Madrid, Spain.; ^3^Molecular Neuroinflammation and Neuronal Plasticity Research Laboratory, Hospital Universitario Santa Cristina, IIS Hospital Universitario de La Princesa, Madrid, Spain.; ^4^Department of Biochemistry, School of Medicine, Universidad Autónoma de Madrid, Spain.; ^5^Nephrology and Hypertension Lab, IIS-Fundación Jimenez Diaz, Madrid, Spain.; ^6^Department of Pharmacological & Toxicological Chemistry, Faculty of Chemical & Pharmaceutical Sciences & Faculty of Medicine, University of Chile, Santiago, Chile.; ^7^Instituto de Investigaciones Biomédicas "Sols-Morreale" IIBM-CSIC-UAM, Madrid, Spain.; ^8^Biomarkers and Personalized Approach to Cancer (BioPAC) Group. Area 3 Cancer -Instituto Ramón y Cajal de Investigación Sanitaria (IRYCIS), Madrid, Spain.; ^9^Biomedical Research Networking Centre on Rare Diseases, CIBERER, ISCIII, Madrid, Spain.; ^10^Department of Medicine, School of Medicine, Universidad Autónoma de Madrid, Madrid, Spain.; ^11^Laboratory of Diabetes and Vascular pathology, IIS-Fundación Jiménez Díaz, Madrid, Spain.; ^12^Biomedical Research Networking Centre on Diabetes and Associated Metabolic Disorders (CIBERDEM), Madrid, Spain.

**Keywords:** SARS-CoV-2 S protein, endothelial cell senescence, cytoprotection, klotho, angiotensin-(1-7)

## Abstract

Premature vascular aging and endothelial cell senescence are major risk factors for cardiovascular diseases and atherothrombotic disturbances, which are main complications of both acute and long COVID-19. The S protein of SARS-CoV2, which acts as the receptor binding protein for the viral infection, is able to induce endothelial cells inflammation and it has been found as an isolated element in the circulation and in human tissues reservoirs months after infection. Here, we investigated whether the S protein is able to directly induce endothelial cell senescence and deciphered some of the mechanisms involved. In primary cultures of human umbilical vein endothelial cells (HUVEC), SARS-CoV-2 S protein enhanced in a concentration-dependent manner the cellular content of senescence and DNA damage response markers (senescence-associated-β galactosidase, γH2AX), as well as growth-arrest effectors (p53, p21, p16). In parallel, the S protein reduced the availability of cytoprotective proteins, such as the anti-aging protein klotho, Nrf2 or heme oxygenase-1, and caused functional harm by impairing *ex vivo* endothelial-dependent vasorelaxation in murine microvessels. These effects were prevented by the pharmacological inhibition of the NLRP3 inflammasome with MCC950. Furthermore, the supplementation with either recombinant klotho or angiotensin-(1-7), equally protected against the pro-senescence, pro-inflammatory and pro-oxidant action of the S protein. Globally, this study proposes novel mechanisms of disease in the context of COVID-19 and its vascular sequelae and provides pharmacological clues in order to prevent such complications.

## INTRODUCTION

Early since the beginning of the COVID-19 pandemia, endothelial injury and thrombotic alterations were reported in patients infected by the SARS-CoV-2 coronavirus. Since then, a large series of clinical reports have identified the vasculature as one of the main trans-organ systems affected by acute SARS-CoV-2 infection [1, 2]. Beyond acute COVID-19 disease, vascular damage and endothelial dysfunction are currently acknowledged to be at the basis of different long-term sequelae following the infection by the coronavirus [3, 4] and thus are gaining importance in the context of post-COVID complications. Premature vascular aging is both a prominent risk for cardiovascular diseases [5] and a biomarker of individual frailty [6]. Among the different hallmarks of aging [7], cellular senescence is one of the main mechanisms contributing to tissue damage. Senescent endothelial cells undergo an irreversible cell cycle arrest and acquire a senescence-associated pro-oxidant and pro-inflammatory secretory phenotype (SASP) that shares many features with the dysfunctional endothelium [8]. The SASP releases cytokines and other inflammatory, pro-thrombotic, and pro-oxidant factors that may alter de cellular redox status and further propagate senescence to neighboring cells thus favoring vascular disease progression [9, 10].

In this context, efforts are being made to better understand the pathophysiological stimuli that promote stress-induced endothelial cell senescence. Improving our knowledge in this field will allow for identifying and validating protective senotherapeutic molecules to prevent the onset of the senescent phenotype in vascular cells. In this context, some molecules like angiotensin (Ang)-(1-7) or klotho, have been revealed as potential anti-senescence compounds [11-13]. Ang-(1-7) is a main heptapeptide of the protective branch of the renin-angiotensin system (RAS). It is generated from its physiological antagonist Ang II by means of angiotensin-converting enzyme-2 (ACE2) activity and exhibits vasodilatory, anti-inflammatory but also anti-senescence activity [13]. In human endothelial cells, Ang-(1-7) upregulates the levels of klotho, a cellular protein also found in its soluble form in the circulation and whose deficiency prompts an accelerated aging phenotype [14, 15]. In vasculature, klotho protects the endothelium against the harmful effects of oxidative stress and promotes the release of nitric oxide thus favoring vasorelaxation [14, 15].

Different studies have underlined the capacity of isolated SARS-CoV-2 elements to directly promote endothelial dysfunction. This is the case for the spike (S) protein, which has evolved during the pandemia affecting the infectivity and/or immunity-escaping ability of the virus and is composed of two main domains, S2 and S1 [16, 17]. The latter contains the receptor binding domain (RBD) that attaches to the host cell receptor, while the S2 domain mediates viral cell membrane fusion and entry [16, 17]. Recently, we demonstrated that the wild-type S protein is itself capable of triggering the priming and activation of the NLRP3 inflammasome, a redox-sensitive first-line component of the innate immune system in human endothelial cells that forms mature interleukin (IL)-1β [18]. However, whether the S protein can directly cause endothelial cell senescence as a mechanism of vascular damage related to SARS-CoV-2 infection requires further attention.

In this study, we addressed the capacity of the isolated SARS-CoV-2 S protein to act as a stressor driving premature senescence in human endothelial cells and deciphered some of the signaling pathways involved in such a deleterious effect, such as the NLRP3 inflammasome, with additional focus on the role of nuclear factor-erythroid 2-related factor 2 (Nrf2), as a major cytoprotective driver. Finally, we tested klotho and Ang-(1-7) as pharmacological tools to prevent such a detrimental action of the SARS-CoV-2 S protein on human endothelial cells.

## MATERIALS AND METHODS

### Materials

M199 culture medium and fetal calf serum (FCS) was from Biological Industries (Beit-Hamek, Israel). Heparin, endothelial cell growth supplement (ECGS), amphotericin, type II collagenase, type I collagen, EDTA, sodium orthovanadate, phenylmethylsulfonyl fluoride (PMSF), noradrenaline (NA; N5785; Sigma; St. Louis, MO, USA), and acetylcholine (ACh; A9101; Merck; Darmstadt, Germany) were purchased from Sigma (St. Louis, MO, USA). Recombinant SARS-CoV-2 wild-type spike (S) protein was purchased from BioTechne (10549-CV; Minneapolis, MN, USA). IL-1β and active human recombinant α-klotho (r-klotho) were purchased from Preprotech (London, UK) and Abcam (ab84072; Cambridge, UK), respectively, while Ang-(1-7) was purchased from Bachem (Bubendorf, Switzerland). The sodium salt CP-456773 (also known as MCC 950) and sulforaphane (SFN) were purchased from Sigma (St. Louis, MO, USA) and LKT Laboratories (Minnesota, USA), respectively.

### HUVEC isolation and culture

Human umbilical vein endothelial cells (HUVEC) were isolated from umbilical cords from donors at Hospital Universitario La Paz (Spain, Madrid) with informed consent, as previously described [15, 19, 20] all procedures followed the Spanish legislation and were under approval of La Paz Hospital Ethics Committee. HUVEC were isolated by chemical digestion with type II collagenase (2 mg/mL), and cultured in M199 medium supplemented with 20 % FCS, 25 μg/mL ECGS, 100 μg/mL heparin, and antibiotics (100 U/mL penicillin, 100 μg/mL streptomycin and 2.5 μg/mL amphotericin B) at 37°C in a humidified atmosphere with 5 % CO2. For all experiments, cells at passages 1-5 were treated with M199 medium supplemented with 10% FCS, ECGS, heparin and antibiotics with the different test compounds for 18-24 h prior to the quantification of the different readouts.

### Senescence-associated β-galactosidase assay

Senescence-associated β-galactosidase (SA-β-gal) staining was performed using a commercial kit from Sigma (CS0030; St. Louis, MO, USA). For each independent experiment, the percentage of SA-β-gal positive cells stained in blue over total cells was determined by blind manual scoring of at least 1,000 cells in 12 randomized fields per treatment, under an inverted microscope Nikon Eclipse T300 (Tokyo, Japan) in phase contrast mode with a 20x objective, as previously described [15, 19, 20].

### DNA foci detection by indirect immunofluorescence

HUVEC cultures were fixed in 4% formaldehyde for 10 min, washed with phosphate-buffered saline (PBS), permeabilized with 0.2% Triton for 5 min and finally blocked with 1% BSA for 1 h. Coverslips were incubated for 1 h with the primary antibody (γH2AX Ser139, Cell Signaling #2595; 1:200) at room temperature, followed by a 1 h incubation with goat-anti-rabbit IgG Alexa Fluor 488 secondary antibody (A-11034, Thermo Fisher Scientific, Illinois, USA). DNA was stained with DAPI. Fluorescence microscopy was performed using a NIKON Eclipse 90i. The image analysis was performed using the software program Nikon NIS-Elements and Image J. Controls without primary antibody, secondary antibody or without both antibodies were used to check specificity of the immunostaining.

### Proteomics

A differential proteomic analysis was performed with HUVEC treated with S protein (35 nM) and its corresponding untreated controls, by means of LC-MS/MS on an Orbitrap Eclipse Tribrid mass spectrometer (Thermo Scientific) using TMT 6-plex isobaric label reagents (ThermoFisher). A total number of 6,145 proteins were quantifiable.

For the representation of the network of statistically significant proteins, String v12.0 was used [21] with a custom value of 0.2 for the interaction score. For Gene Ontology analysis, ShinyGO v0.77 [22] with GO biological process and FDR of 0.2 and Metascape. v3.5.20240101 [23] with enrichment in GO biological process with a p-value of 0.05, were used. A more detailed version of this section is provided in Supplementary Material.

### Western blotting

For protein quantification by Western Blot, HUVEC were lysed and the protein content in cell lysates was quantified by the bicinchoninic acid (BCA) method (Thermo Fisher Scientific, Illinois, USA). Thereafter, 20 μg of protein lysates were separated by SDS-PAGE electrophoresis and transferred to polyvinyl membranes (Merck, Darmstadt, Germany), as previously described [15]. Primary antibodies against γH2AX (2577S; Cell Signaling Technology, USA; 1:1000), p53 (sc-126; Santa Cruz Biotechnology, USA, 1:1000), p21 (sc-6246; Santa Cruz Biotechnology, USA; 1:500), p16 (550834; BD Pharmigen, USA;1:1000), heme oxygenase-1 (HO)-1 (sc-136960; Santa Cruz Biotechnology, USA; 1:1000), klotho (sc-515939; Santa Cruz Biotechnology; 1:1000) and Nrf2 (sc-518033; Santa Cruz Biotechnology, USA; 1:500) were used, followed by incubation with corresponding horseradish peroxidase-conjugated secondary antibodies, i.e, goat anti-mouse IgG (AP127P; Merck, Darmstadt, Germany; 1:25,000) or goat anti-rabbit IgG (H + L)-HRP conjugate (1706515; Bio-Rad; California, USA; 1:10,000). Protein levels were normalized to β-actin signal (Sigma-Aldrich; 1:10,000). Immunoreactive bands were detected using an enhanced chemiluminescence ECL detection kit (1705062; Bio-Rad; California, USA) and quantified by densitometry using ImageJ 1.51w free software.

### Total RNA isolation and quantitative real-time (qRT)-PCR

For gene expression analysis, total RNA was extracted from HUVEC using TRIZOL-Chloroform method with NZYol (NZYTech, Lisbon, Portugal). RNA integrity was tested by a NanoDrop 2000 spectrophotometer (Thermo Fisher Scientific; Illinois, USA), and cDNA synthesis was performed using the Maxima H Minus First Strand cDNA Synthesis Kit (K1652; Thermo Fisher Scientific; Illinois, USA), with 2 µg of RNA as template and following the manufacturer’s instructions. qRT-PCR reactions for HO-1 and klotho were performed with iTaq Universal SYBR Green Supermix (Bio-Rad; California, USA) on a Thermo ABI QuantStudio 5 Real-Time PCR System (Thermo Fisher Scientific; Illinois, USA) and specific primers (Sigma; St. Louis, MO, USA) against HO-1, klotho, and 18S (see primers in Supplementary methods Table 1). IL-6 (Hs00985639_m1), MCP-1 (Hs00234140_m1) and TNF-α (Hs00174128_m1) were analyzed using predeveloped Taqman primers (Applied Biosystems, Foster City, CA). The qPCR conditions were 95 ºC for 10 min followed by 40 cycles of 95 ºC for 15 s and 60 ºC for 1 min. The quantification of gene expression relative to the housekeeping gene 18S was determined by 2^-ΔΔCt^ method and normalized to the untreated control.

**Figure 1.**
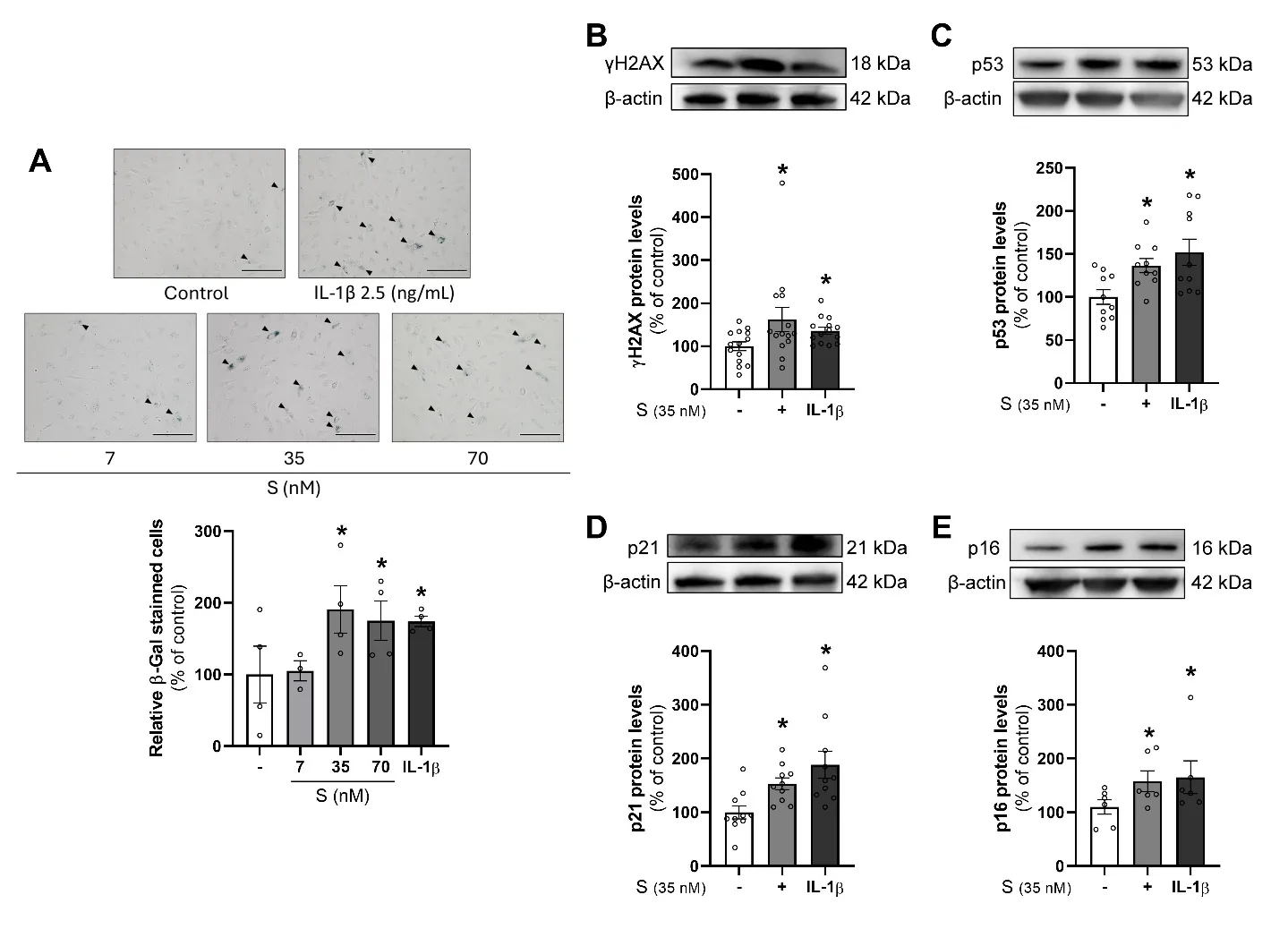
**SARS-CoV-2 S protein induces cellular senescence in endothelial cells**. Human umbilical vein endothelial cells (HUVEC) were treated for 24 h with S protein (S; 7, 35, or 70 nM), or IL-1β (2.5 ng/mL) after which (**A**) SA-β-Gal staining was performed and quantified (*n*=4, except *n*=3 for S 7 nM). Black arrows point to positive SA-β-gal-stained cells in a representative experiment. Scale bar represents 150 µm. Similarly, after exposure to S protein (35 nM) or IL-1β (2.5 ng/mL) for 18 h, the protein levels of senescence markers such as (**B**) γH2AX histone (*n*=11), (**C**) p53 (*n*=10), (**D**) p21 (*n*=10) and (**E**) p16 (*n*=7, except *n*=6 for S 35 nM) were determined by Western blot. Representative gels are shown on top of the corresponding graphs, with β-actin used as a loading control. All bar graphs represent the mean ± SEM. Statistical differences were tested with t-test (**A, C, D**) or Mann-Whitney (**B, E**). * p < 0.05 versus control.

### Microvascular reactivity

For reactivity experiments, 3-month-old female C57BL/6J mice were used. Segments from first branch mesenteric arteries (internal diameter 180-300 μm) were mounted on a small vessel myograph (DMT, Denmark) to measure isometric tension, and maintained in Krebs-Henseleit solution at physiologic conditions (37ºC, continuous bubbling with 95 % O2 - 5 % CO2 mixture and pH 7.4), as described before [15, 24]. Arteries were contracted with 2 μM noradrenaline (NA) and then the vasoactive responses to cumulative concentrations of acetylcholine (ACh; 0.1 nM to 10 μM) were tested. In some experiments, the mesenteric segments were pre-incubated for 1 h with the S protein (35 nM) alone or in combination with MCC950 (10 µM), r-klotho (1 nM) or Ang-(1-7) (100 nM). In order to address endothelium-independent vasorelaxation, concentration-dependent curves to sodium nitroprusside (SNP; 10 nM to 3 mM) were also performed. The reactivity protocol is summarized in the Supplementary Figure 1.

### Statistical analysis

Data are expressed as mean ± standard error of the mean (SEM) for the indicated numbers of independent experiments. Statistical analysis was performed using GraphPad, Prism 8.0.2 software (California, USA). Normal distribution was tested with Shapiro-Wilk test. Statistical differences were analyzed by t-test for normal distributed data and Mann-Whitney test for non-normal distribution. For the large sample size in the DNA foci detection by indirect immunofluorescence, we used Kolmogorov-Smirnov for normal distribution analysis and Kruskal-Wallis to test the differences between groups. For the comparison of continuous variables in the vascular reactivity experiments statistically significant differences were analyzed with two-way ANOVA. A p-value ≤ 0.05 was considered statistically significant.

**Figure 2.**
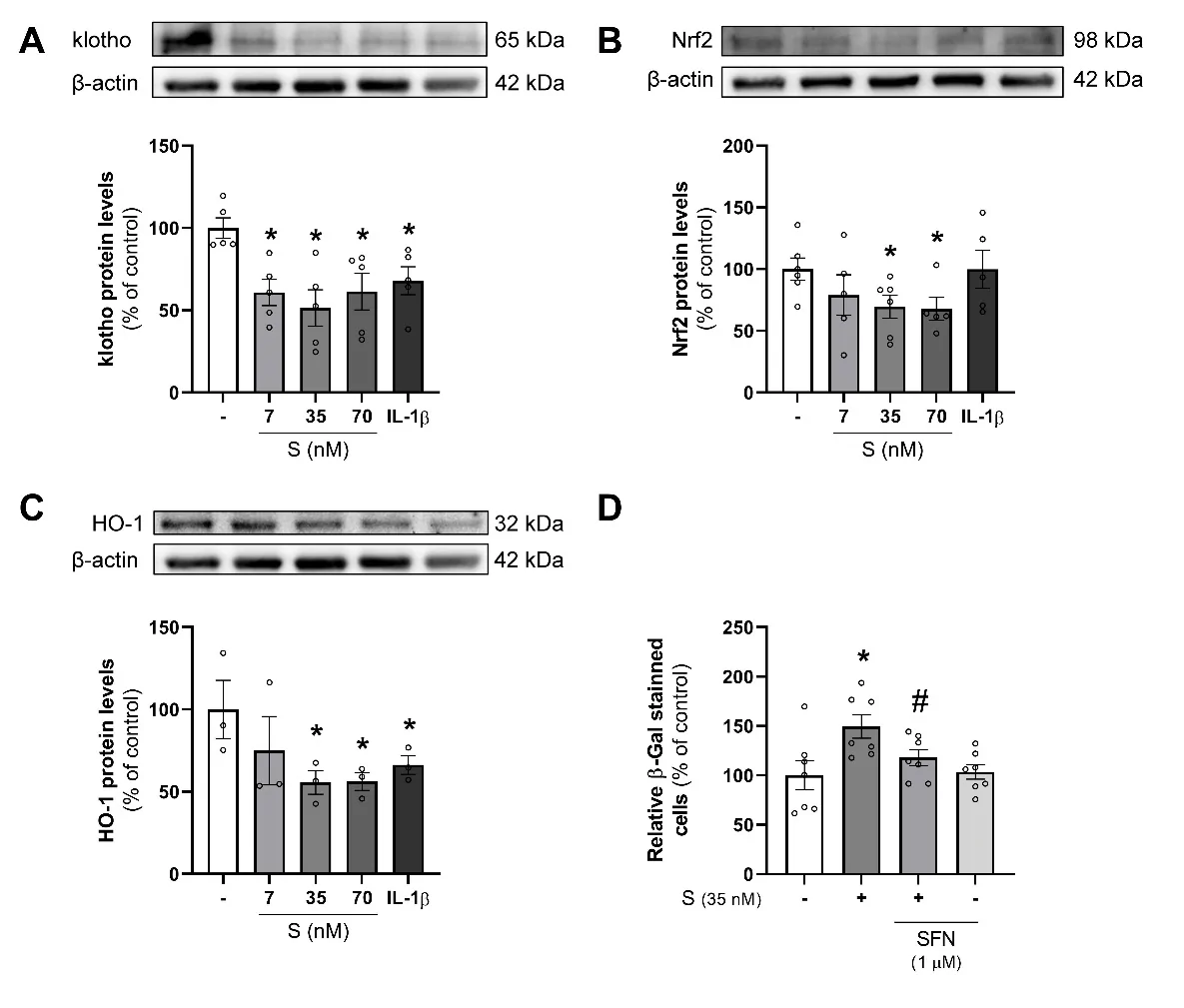
**SARS-CoV-2 S protein reduces cellular antioxidant and anti-senescence defenses**. Human umbilical vein endothelial cells (HUVEC) were treated for 24 h with S protein (S; 7, 35, or 70 nM), or IL-1β (2.5 ng/mL) for 18 h after which the protein levels of (**A**) klotho (*n*=5), (**B**) Nrf2 (*n*=5, except *n*=6 for untreated control and S 35 nM) and (**C**) heme oxygenase-1 (HO-1) (*n*=3) were determined by Western blot. (**D**) In another set of experiments SA-β-gal staining (*n*=7) was performed in HUVEC stimulated for 24 h S (35 nM) with or without the Nrf2 activator sulforaphane (SFN; 1 µM) for 24 h. Representative gels are shown on top of the corresponding graphs, with β-actin used as a loading control. All bar graphs represent the mean ± SEM. Statistical differences were tested with t-test (**B, C, D**) or Mann-Whitney (**A**). * p < 0.05 versus control. # p< 0.05 versus S protein.

## RESULTS

### SARS-CoV-2 S protein elicits senescence in human endothelial cells

In HUVEC cultures exposed to increasing concentrations of S protein (7, 35 and 70 nM), an enhancement of the relative number of cells positively stained for SA-β-gal (SA-β-gal^+^) was observed from a threshold concentration of 35 nM (Fig. 1A). This concentration was then chosen for subsequent experiments with S protein. A differential abundance proteomic analysis was performed comparing S protein treated HUVEC (35 nM) versus untreated cells. The differential proteins obtained were subjected to a Gene Ontology enrichment analysis using ShinyGO and Metascape softwares, which highlighted, among other, the terms “DNA damage response” and “regulation of signal transduction by p53 class mediator” supporting the results of the SA-β-gal assay (Supplementary Fig. 2A to 2C).

Double strand DNA damage prompts the so-called DNA-damage response (DDR), which is considered a major event triggering pro-senescence responses and growth arrest [25]. Figure 1B shows that S protein (35 nM) enhanced the levels of γH2AX histone, a marker and effector of the DDR. This result was further reinforced by the quantification of the number of DNA damage foci using immunofluorescence techniques (Supplementary Fig. 3). In parallel, S protein augmented the levels of the downstream effector proteins p53, p21 and p16, which eventually lead to the growth arrest characteristic of cellular senescence (Fig. 1C to 1E). All these pro-senescent effects of the viral protein were shared by the cytokine IL-1β, that was used as a positive control for inducing endothelial cell senescence, as previously reported by us and others [15, 19] (Fig. 1A to 1E).

**Figure 3.**
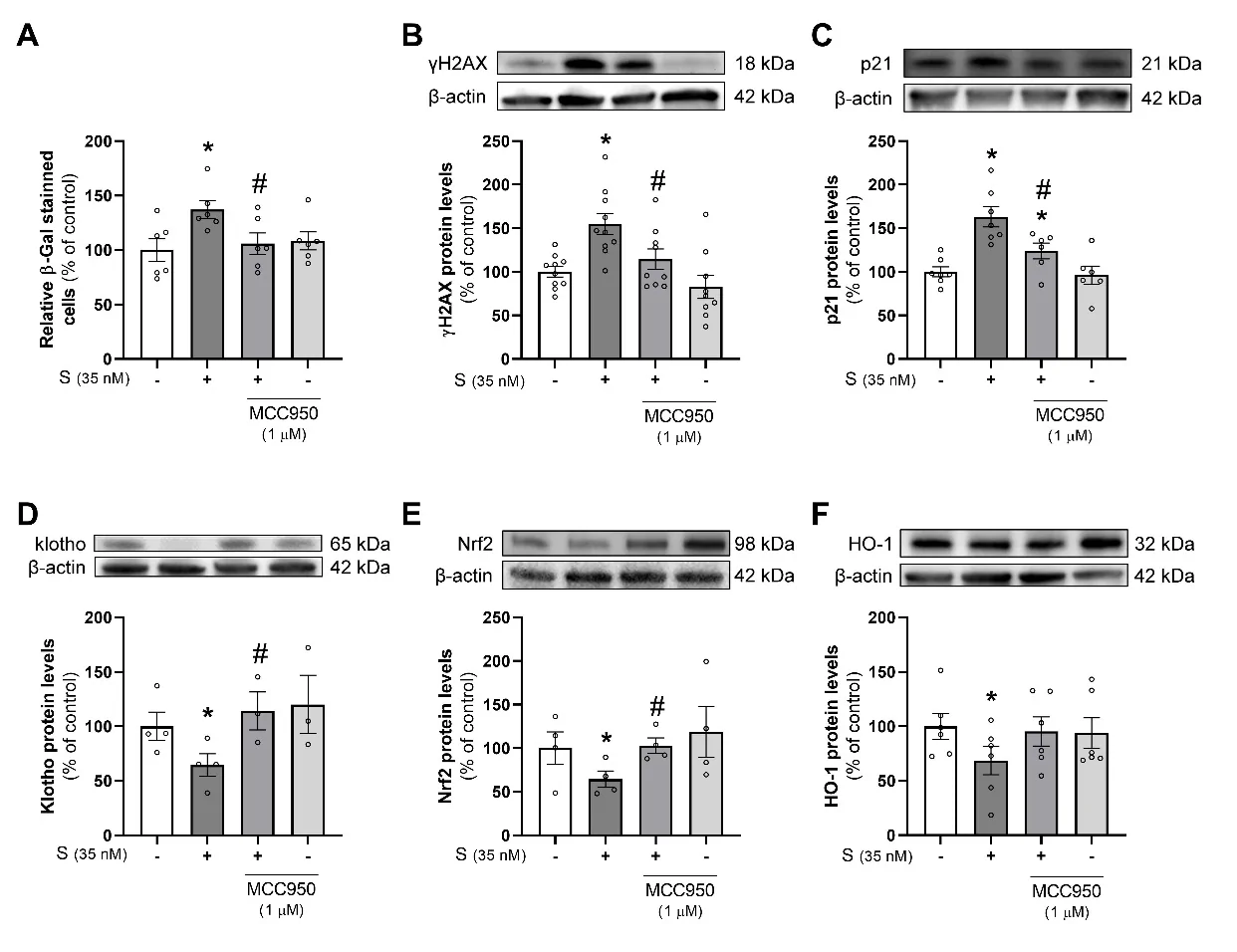
**NLRP3 inflammasome inhibition prevents the decrease in cytoprotective proteins and cellular senescence induced by SARS-CoV-2 S protein**. Human umbilical vein endothelial cells (HUVEC) were treated for 18-24 h with S protein (S, 35 nM), MCC950 (1 µM) or both. Thereafter, (**A**) SA-β-gal staining (*n*=6) was performed and the protein levels of (**B**) γH2AX histone (*n*=10, except *n*=9 for S + MCC950 and MCC950 alone), (**C**) p21 (*n*=7, except *n*=6 for S + MCC950 and MCC950 alone), (**D**) klotho (*n*=4, except *n*=3 for S + MCC950 and MCC950 alone), (**E**) Nrf2 (*n*=4) and (**F**) heme oxygenase-1 (HO-1) (*n*=6) were determined by Western blot (WB). Representative gels are shown on top of the corresponding graphs for WB with β-actin used as a loading control. All bar graphs represent the mean ± SEM. Statistical differences were tested with t-test (**A, B, C, D, E**) or Mann-Whitney (**F**). * p < 0.05 versus control. # p < 0.05 versus to S protein.

### SARS-CoV-2 S protein induces the SASP phenotype

The SASP is a feature of senescent cells that facilitates the release of a series of pro-inflammatory and chemotactic molecules mediating the recruitment of immune cells [8]. We have recently shown that HUVEC exposed to S protein (35 nM) exhibit increased IL-1β synthesis and release [18]. Here we demonstrate an increased expression of other SASP components, such as IL-6, tumor necrosis factor (TNF)-α and monocyte chemoattractant protein (MCP)-1 elicited by the S protein (Supplementary Fig. 4A to 4C), further confirming the direct induction of a secretory phenotype by this isolated viral element.

### S protein reduces the levels of anti-aging and anti-oxidant protective proteins

In order to deepen into the mechanisms of endothelial senescence induction by the S protein, we next explored a role for the exhaustion of intracellular defense proteins. A significant decrease in the cellular content of the anti-aging protein klotho was observed in HUVEC cultures exposed to the S protein (7 to 70 nM) (Fig. 2A). This was paralleled by diminished intracellular levels of the antioxidant and anti-inflammatory protein heme-oxygenase (HO)-1 and its main transcriptional driver, Nrf2 (Fig. 2B and 2C). In fact, the administration of sulforaphane (1 µM), a direct Nrf2 activator, prevented the increase in SA-β-gal^+^ endothelial cells induced by the SARS-CoV-2 S protein (Fig. 2D).

**Figure 4.**
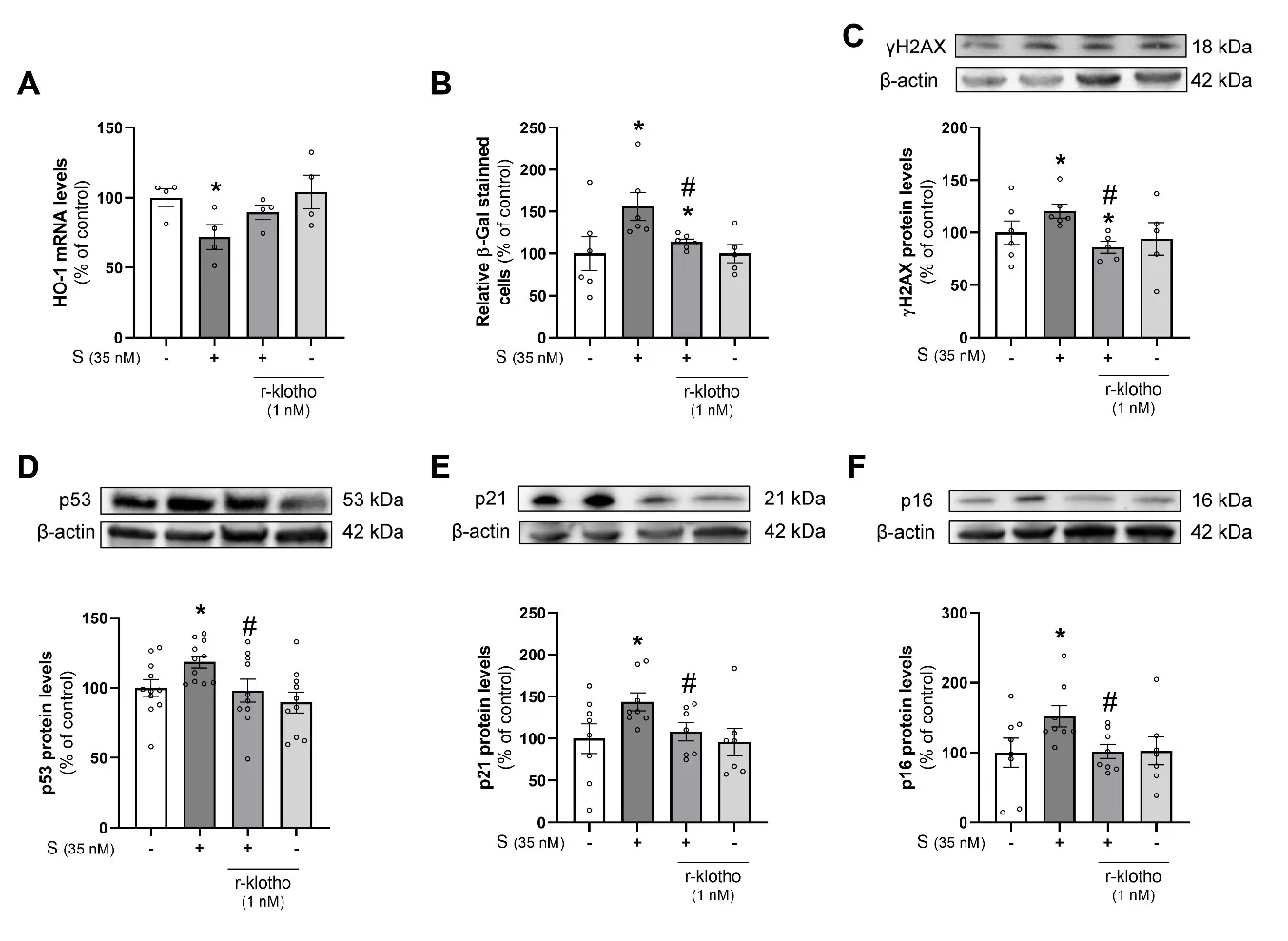
**r-klotho prevents deficient cytoprotection and endothelial cell senescence induced by SARS-CoV-2 S protein**. Human umbilical vein endothelial cells (HUVEC) were treated for 18-24 h with S protein (S, 35 nM), r-klotho (1 nM) or both. Afterwards, (**A**) heme oxygenase-1 (HO-1) expression (*n*=4) was determined by RT-qPCR using 18S housekeeping gene for normalization, (**B**) SA-β-gal staining (*n*=6, except *n*=5 for r-klotho alone) was performed and protein levels of (**C**) γH2AX histone (*n*=6, except *n*=5 for S + r-klotho and r-klotho alone), (**D**) p53 (*n*=11, except *n*=10 for S + r-klotho and r-klotho alone), (**E**) p21 (*n*=8, except *n*=7 for S + r-klotho and r-klotho alone) and (**F**) p16 (*n*=8, except *n*=7 for r-klotho alone) were determined by Western blot (WB). Representative gels are shown on top of the corresponding graphs for WB with β-actin used as a loading control. All bar graphs represent the mean ± SEM. Statistical differences were tested with t-test (**B, C, D, F**) or Mann-Whitney (**A, E**). * p < 0.05 versus control. # p < 0.05 versus to S protein.

### NLRP3 inflammasome blockade prevents S protein-induced cellular senescence and antioxidant defense reduction

Sustained inflammation is a critical component of aging tissues, including the vascular one [26]. In previous studies we have shown that the activation of the NLRP3 inflammasome plays a critical role in the induction of endothelial cell senescence by several extracellular stressors such as adipokines or IL-1β [19, 20, 24]. Moreover, we have recently reported the capacity of the S protein to both prime and activate the NLRP3 inflammasome [18]. Here we demonstrated a role of the NLRP3 inflammasome in the endothelial senescence induced by the S protein by using MCC950 (1 µM), a specific blocker of NLRP3 inflammasome assembly and activation. In fact, MCC950 prevented not only the increase in SA-β-gal^+^ endothelial cells (Fig. 3A), but also the enhancement of γH2AX histone and p21 protein levels stimulated by SARS-CoV-2 S protein (Fig. 3B and 3C). Importantly, MCC950 equally prevented the reduction in klotho, Nrf2 and HO-1 protein levels, thus showing a causative link between the NLRP3 inflammasome activation and the decline of cellular antioxidant and anti-inflammatory defenses (Fig. 3D to 3F).

### r-klotho and Ang-(1-7) supplementation prevents endothelial cell senescence induced by SARS-CoV-2 S protein

We next assessed whether the pharmacological supplementation with exogenous r-klotho (1 nM) could prevent the endothelial cell senescence induced by the S protein. r-klotho blunted the deficient HO-1 expression triggered by the viral protein (Fig. 4A). In parallel, r-klotho restrained the induction of SA-β-gal^+^ cells (Fig. 4B) and the enhancement of γH2AX, p21, p53, and p16 protein levels by the viral S protein, without affecting basal parameters by itself (Figu. 4C to 4F). In addition, the enhanced number of γH2AX foci was also prevented with r-klotho (Supplementary Fig. 3).

**Figure 5.**
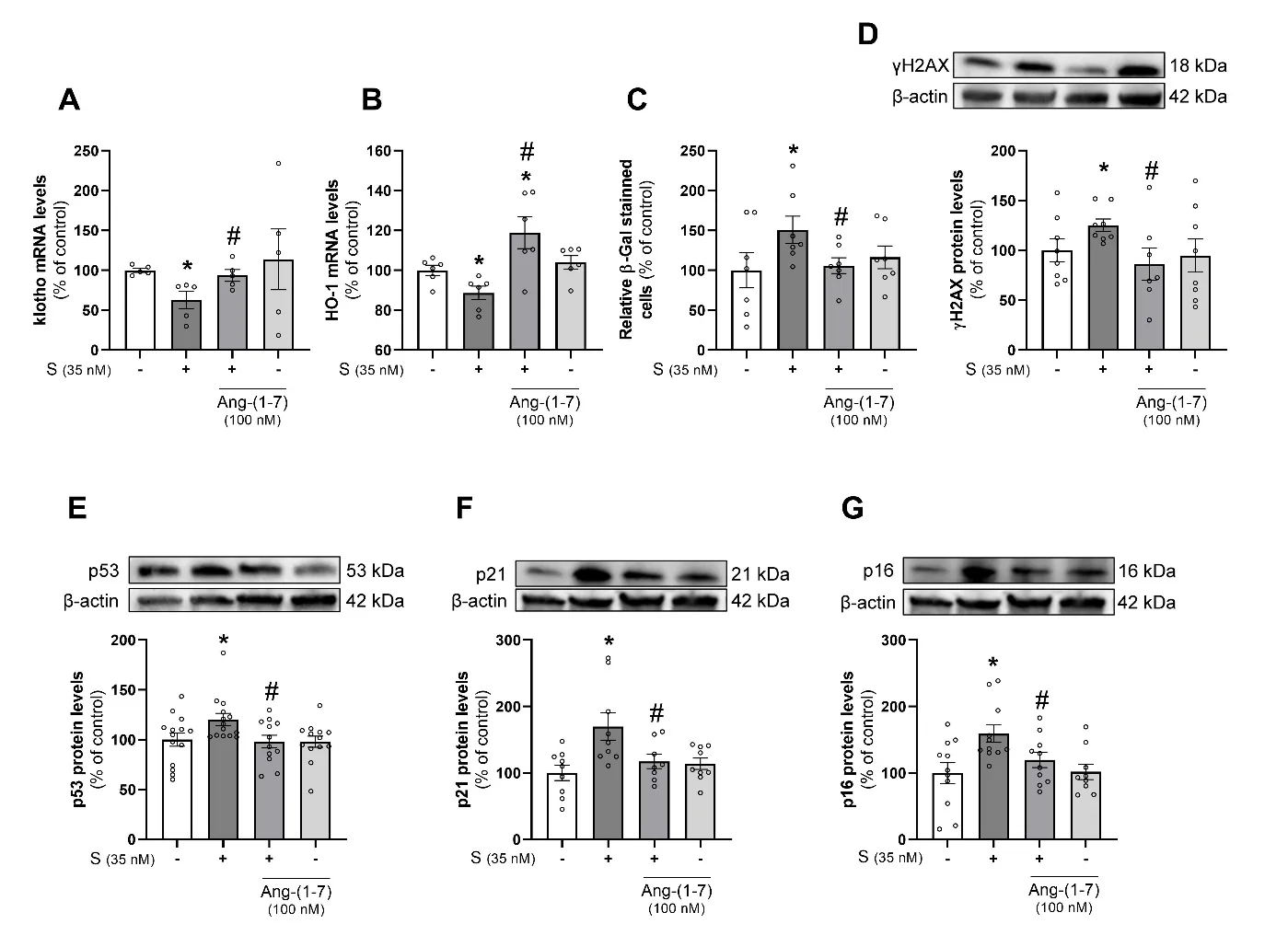
**Ang-(1-7) prevents deficient cytoprotection and endothelial cell senescence induced by SARS-CoV-2 S protein**. Human umbilical vein endothelial cells (HUVEC) were treated for 18-24 h with S protein (S; 35 nM), Ang-(1-7) (100 nM) or both. Afterwards, (**A**) klotho (*n*= 5) and (**B**) heme oxygenase-1 (HO-1) expression (*n*=6) were determined by RT-qPCR using 18S housekeeping gene for normalization, (**C**) SA-β-gal staining (*n*=7) was performed and protein levels of (**D**) γH2AX histone (*n*=8, except *n*=7 for S + Ang-(1-7)), (**E**) p53 (*n*=14, except *n*=12 for S + Ang-(1-7) and *n*= 13 for Ang-(1-7) alone), (**F**) p21 (*n*=9, except *n*=8 for S + Ang-(1-7)) and (**G**) p16 (*n*=11, except *n*=9 for Ang-(1-7) alone and *n*=10 for S + Ang-(1-7)) were determined by Western blot (WB). Representative gels are shown on top of the corresponding graphs for WB with β-actin used as a loading control. All bar graphs represent the mean ± SEM. Statistical differences were tested with t-test (**A, B, F, G**) or Mann-Whitney (**C, D, E**). * p < 0.05 versus control. # p < 0.05 versus to S protein.

We have previously shown that Ang-(1-7), a key component of the protective branch of the renin-angiotensin system (RAS), is capable of augmenting klotho levels in HUVEC [15]. In accordance with this previous finding, we observed that Ang-(1-7) prevented the defective expression of both klotho (Fig. 5A) and HO-1 (Fig. 5B) induced by the S protein and was equally capable to prevent endothelial cell senescence triggered by this isolated viral element, without affecting the basal levels of the different markers studied by itself (Fig. 5C to 5G and Supplementary Fig. 2). Ang-(1-7) also prevented the induction of IL-6, TNF-α and MCP-1 mRNA levels by S protein (Supplemental Fig. 4A to 4C).

**Figure 6.**
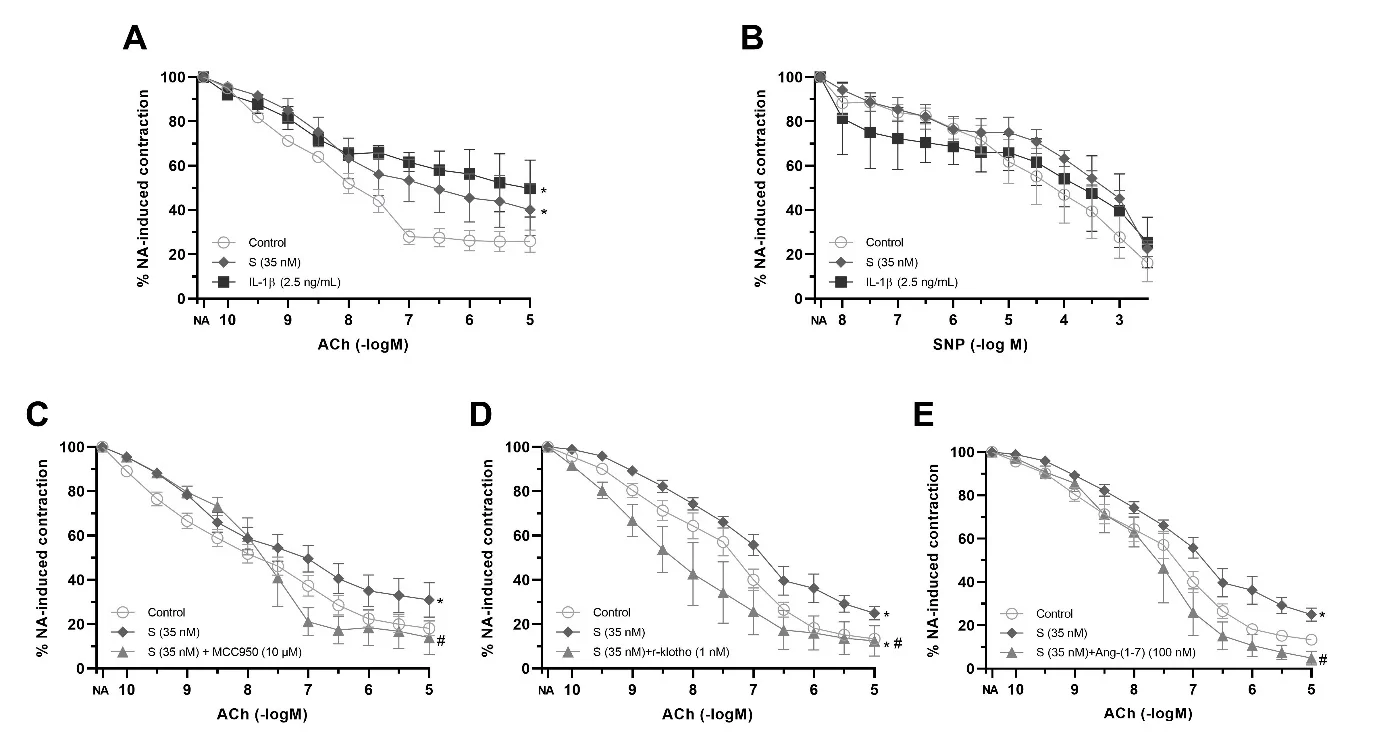
**The endothelium-dependent relaxation impaired by SARS-CoV-2 S protein is prevented by NLRP3 inflammasome blockade or the supplementation with r-klotho or Ang-(1-7)**. Isolated mesenteric microvessels from C57BL/6J mice were preincubated for 1 h with IL-1β (2.5 ng/mL) or S protein (35 nM) after which vascular relaxations in response to increasing concentrations of (**A**) acetylcholine (ACh; 0.1 nM to 10 μM), *n*=5-7 segments from 5 mice; or (**B**) sodium nitroprusside (SNP; 10 nM to 3 mM), *n*=3-5 segments from 5 mice; were assessed. In selected experiments, (**C**) the inflammasome inhibitor MCC 950 (10 µM), *n*=4-19 segments from 4-8 mice; (**C**) r-klotho (1 nM), *n*=4-12 segments from 4 mice; or (**D**) Ang-(1-7) (100 nM), *n*=3-12 segments from 3-4 mice; were added during the preincubation period. Results are presented as the percentage of contraction to noradrenaline (NA; 2 µM). More details on the exact number of mice and segments per treatment can be found in Supplementary Tables 2-5. Data are represented as mean ± SEM. Statistical differences were tested with two-way ANOVA. * p<0.05 versus control; # p<0.05 versus S protein.

### S protein induces microvascular endothelial dysfunction *ex vivo*

Endothelial cell senescence is functionally associated with features of endothelial dysfunction, including altered vascular reactivity and defective vasorelaxation [27]. Using *ex vivo* murine microvascular mesenteric segments, we observed that S protein impaired the endothelium-dependent relaxation triggered by cumulative concentrations of ACh (0.1 nM to 10 µM), although to a lesser extent than the cytokine IL-1β (2.5 ng/mL) (Fig. 6A). On the contrary, exposing the microvessels to S protein did not alter the endothelium-independent vasorelaxation induced by sodium nitroprusside (SNP; 10 nM to 3 mM) (Fig. 6B). The defective vasorelaxation induced by the S protein was prevented by preincubating the vessels with the NLRP3 inhibitor MCC950 (10 µM) (Fig. 6C) as well as by supplementing with r-klotho (1 nM) or Ang-(1-7) (100 nM) (Fig. 6D and 6E).

## DISCUSSION

Early since the beginning of the COVID-19 pandemia endothelial dysfunction was identified as one of the major complications of acute SARS-CoV-2 infection, making the vessels more prone to inflammation and coagulation abnormalities [3, 28]. Currently, clinical evidence has unveiled that vascular abnormalities are also major players in the context of long-COVID [3]. Indeed, different biomarkers related to endothelial dysfunction have been found altered in the circulation of long-COVID patients with persistent symptoms even months after acute infection [29]. Since blood vessels transverse every organ, vascular abnormalities can directly contribute to the wide and complex array of dysfunctionalities and symptoms of COVID-19. Understanding vascular complications in COVID-19 is thus a complex but necessary task to provide effective therapeutical approaches.

Recent hypotheses have raised the question whether the SARS-CoV-2 S protein as an isolated element can be responsible, at least in part, for the complications of long COVID-19 [30]. Persistent circulating S protein levels have been found in patients with post-acute COVID in association with a range of sequelae, including cardiovascular ones [31]. In this study we demonstrate that SARS-CoV-2 wild-type S protein can provoke *per se* premature senescence in primary cultures of human endothelial cells, as well as a significant vascular dysfunction in terms of endothelial-dependent vasorelaxation. This is in line with other studies reporting biological activities of the isolated S protein in terms of endothelial activation [32], cardiac pericyte disturbance [33] or blood-brain barrier integrity disruption [34], among others.

**Figure 7.**
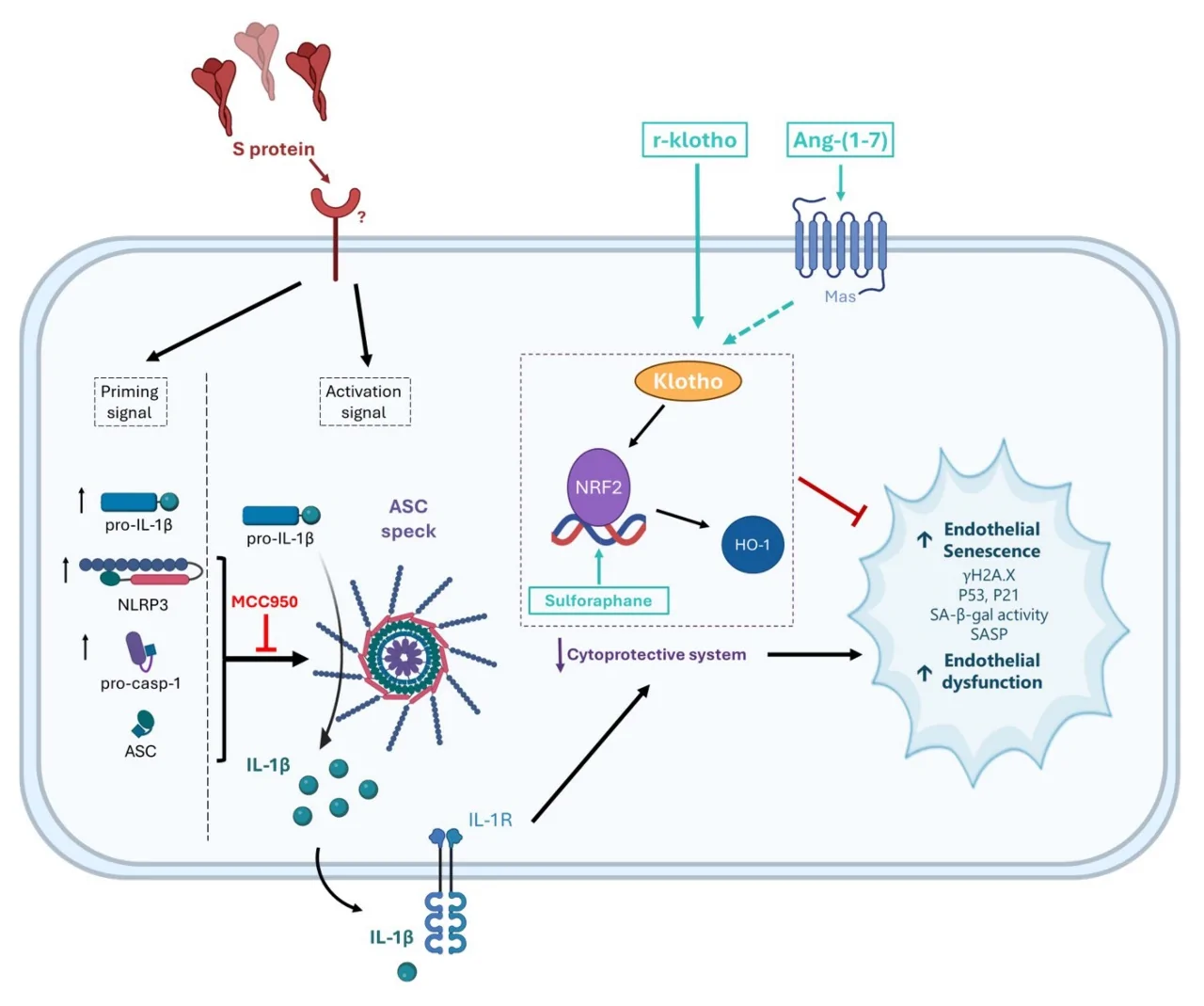
**Graphical abstract summarizing the direct actions of SARS-CoV-2 S protein reported in the present study**. SARS-CoV-2 S protein promotes by itself human endothelial cell senescence and DNA damage response, determined by the induction of markers such as senescence-associated β-galactosidase (SA-β-gal) or histone γH2AX and growth-arrest effectors like p53, p21 and p16. This effect is paralleled by *ex vivo* endothelial dysfunction in terms of impaired endothelial-dependent relaxation of murine microvessels. Such deleterious effects of the isolated viral protein are associated to a reduction of anti-aging and cytoprotective proteins, such as klotho, Nrf2 and heme oxygenase-1 (HO-1). In fact, activating Nrf2 with the drug sulforaphane or supplementing with recombinant klotho (r-klotho) or the klotho inducer angiotensin (Ang)-(1-7) prevents the pro-senescence action and the defective vasorelaxation induced by SARS-CoV-2 S protein. Moreover, blocking the activation of the NLRP3 inflammasome by means of MCC950 protects against the restricted cytoprotective protein availability, cellular senescence and microvascular dysfunction directly caused by SARS-CoV-2 S protein.

Interestingly, a number of recent studies have reported very low or no levels of ACE at the surface of endothelial cells and a very limited productive replicative capacity of SARS-CoV-2 in this cell type [18, 35]. This reinforces a role for other viral mechanisms, including isolated SARS-CoV-2 elements, in eliciting endothelial damage and dysfunction. Through which receptors may the S protein exert its direct action on the endothelium remains to be better defined, although candidates besides ACE2 have been proposed, such as toll-like receptors 4, which in our hands was not effective [18] dipeptidyl-peptidase 4 or basigin-CD147, among other [36, 37]. Taken together, these observations highlight the extreme complexity of the actions exerted by the SARS-CoV-2 coronavirus or its isolated elements.

In the blood vessels, the accumulation of senescent endothelial cells, which are particularly abundant in human atherosclerotic lesions [38], is associated with the development and progression of vascular dysfunction. By the release of SASP components, such as IL-6 and other or IL-1β, endothelial senescent cells contribute to creating a pro-oxidant and pro-inflammatory environment that further expands senescence and drives vascular damage [39]. Moreover, endothelial senescent cells are known to over-express substances such as plasminogen activator inhibitor and von Willebrand factor favoring a thrombogenic environment and clotting [40, 41], which is one of the main features of both acute and persistent COVID-19 [28, 39].

Aging is associated with a decline in intracellular defense systems, while a prominent feature of endothelial cells senescence is impaired redox homeostasis with reduced antioxidant capacity [39]. Among the main factors that drive cellular protection, Nrf2 is a major evolutionary conserved cytoprotective system, which is nowadays considered a powerful modulator of species longevity [42] and a molecular link between oxidative stress regulation and aging [43]. Nrf2 responds against oxidant challenges by promoting the expression of genes encoding for antioxidant and anti-inflammatory proteins, among which HO-1 provides cell protection by degrading the pro-oxidant heme and ultimately forming bilirubin together with the signaling gas carbon monoxide [42]. Here, we unveil the capacity of the SARS-CoV-2 S protein to weaken such an essential protective system in human endothelial cells.

An explanation for such a deleterious effect may reside in the lower cellular levels of the klotho anti-aging protein achieved in the presence of the viral S protein. In endothelial cells, klotho is an inducer of Nrf2 [44] that protects against the harmful effects of oxidative stress while exerting anti-apoptotic and anti-senescence properties [15, 45]. Accordingly, supplementation of human endothelial cultures with r-klotho prevented the pro-senescence effect of the viral S protein. Equally, the addition of Ang-(1-7) showed defensive effects, which can be explained since this pivotal component of the protective branch of the RAS enhances the intracellular levels of klotho and thus of Nrf2 and HO-1 in endothelial cells, as previously described [15]. Moreover, both r-klotho and Ang-(1-7), which favor the release endothelial nitric oxide [46-48], were capable to blunt the defective endothelium-dependent vasorelaxation, one of the earliest markers of vascular dysfunction and disease, elicited by the viral S protein in isolated murine microvessels. Thus, in addition to preventing endothelial cell senescence induced by Ang II or RAS-independent components, such as IL-1β [15], r-klotho and Ang-(1-7) reveal as compounds with a more ample spectrum and capable to equally prevent the pro-senescence action of isolated viral particles. Altogether, these observations sustain a role for both molecules in the emerging field of senotherapeutic drugs. Rather than acting as senolytics or senescent cell killers, both compounds would behave as senostatic drugs capable of preventing the onset of endothelial cell senescence and the subsequent release of pro-inflammatory SASP components.

Indeed, we have recently demonstrated the capacity of S protein to prime and activate NLRP3 inflammasome in human endothelial cells, which is in turn related to an over-expression of endothelial pro-coagulant factors [18]. Here we demonstrate that the activation of such a first-line component of the innate immune system also mediates the exhaustion of cytoprotective defense and premature human endothelial cells senescence triggered by the S protein. Globally, the activation of the NLRP3 inflammasome arises a central mechanism in the deleterious action of SARS-CoV-2 S protein in the human endothelium. In fact, recombinant IL-1β was able to mimic most of the actions of the S protein. This is of pathophysiological and therapeutical importance, since IL-1β, a major product of the NLRP3 inflammasome, has been directly involved in human vascular disease at the light of the clinical trial CANTOS [49]. In fact, SARS-CoV-2 has been shown to trigger the IL-1/IL-6 pathway to a larger extent than other coronaviruses [50]. Moreover, NLRP3 inflammasome products, such as IL-1β and IL-18, are increased in patients with severe acute COVID-19 and positively correlated with adverse clinical outcomes [51].

Overall, the present findings reveal that persisting isolated elements of SARS-CoV-2, such as the S protein of the viral crown, can trigger pro-inflammatory and pro-oxidant harm in the vasculature favoring premature endothelial cell senescence and dysfunction (Fig. 7). Importantly, these observations provide therapeutic clues to dampen such a deleterious action on the vasculature. Blocking the over-activation of the NLRP3 inflammasome and the excessive generation of its end-products or supplementing with drugs, such as r-klotho or Ang-(1-7), capable to restore the cytoprotective capacity of the endothelium should be considered pharmacological options to attenuate potential vascular sequelae derived from COVID-19.

## Supplementary Materials

The Supplementary data can be found online at: www.aginganddisease.org/EN/10.14336/AD.2024.0405.
