# Supplementary material for: SARS-CoV-2 S Protein Reduces Cytoprotective Defenses and Promotes Human Endothelial Cell Senescence
Source: Aging Dis. 2024 Jun 25;16(3):1626–38. doi: 10.14336/AD.2024.0405 (PMC12096926; doi:10.14336/AD.2024.0405)
Supplement: Supplementary file 1 — The Supplementary data can be found online at: www.aginganddisease.org/EN/10.14336/AD.2024.0405. [file AD-16-3-1626-s.pdf]

SUPPLEMENTARY DATA

SARS-CoV-2 S Protein Reduces Cytoprotective Defenses and Promotes Human Endothelial Cell Senescence

Alicia Villacampa, Licia Shamoon, Inés Valencia, Cristina Morales, Sofía Figueiras, Fernando de la Cuesta, Dolores Sánchez-Niño, Guillermo Díaz-Araya, Isabel Sánchez-Pérez, Óscar Lorenzo, Carlos Félix Sánchez-Ferrer, Concepción Peiró

© 2024. Villacampa A. et al. Published online at http://www.aginganddisease.org/EN/10.14336/AD.2024.0405

SUPPLEMENTARY DATA

Supplementary Materials

Supplemental Methods

Proteomics

HUVECs treated with S protein (35 nM) and its corresponding untreated controls (*n*=4 per group) were disrupted in 300 µL of lysis buffer (2.2 % SDS, 0.1 M Tris-HCl, pH 7.5) with an ultrasonic processor VibraCell VCX 130PB (Sonics&Materials) using the following parameters: 40 % amplitude; 10 x 1 s pulse; tubes kept on ice. Cell lysates were then heated at 95º C for 5 min. After being tempered, lysates were sonicated again (5 x 1 s pulse) and centrifuged at 16000 g at 13 °C for 20 min. These two steps were repeated to ensure a good cell disruption and to obtain clarified lysates to be used in further analysis. Protein abundance was quantified by the microBCA method. In all cases, the coefficient of variation (CV) among sample triplicates was lower than 6 %. Aliquots from each cell lysate were kept at -40 °C until use. Cell lysates were digested by the Filter-Aided Sample Preparation (FASP) method using 10 kDa Amicon centrifugal filters (Millipore). Eluted tryptic peptides were tagged with TMT 6-plex isobaric label reagents (ThermoFisher) and 6-plex mixes were cleaned-up with C18 columns (Agilent). Afterwards, High pH Reverse phase (HP-RP) liquid chromatography with 2 min fraction collection was conducted to separate labelled peptides on an Evosep One (EV-1000, Evosep) chromatographic system. From each fraction 30 µL were taken for LC-MS/MS analysis on an Orbitrap Eclipse Tribrid mass spectrometer (Thermo Fisher Scientific, Illinois, USA) coupled to the chromatographic system.

A total number of 73,581 PSMs were identified after database search with Proteome Discoverer 1.4. Once those assigned to more than one protein group were filtered, 70,794 PSMs remained. The quantification of the proteins was carried out based on the intensity of the reporter ions derived from the TMT labelling. The TMT intensity of the reporter ions was normalized using the total abundance of each TMT-label to minimize the error due to different protein load of each channel. A total number of 6,145 proteins were quantifiable. Statistical analysis to determine differential proteins and peptides was performed using DanteR software (http: //omics.pnl.gov/software/danter). Once loaded into DanteR software, missing values were imported, and data-quantiles normalization was performed.

Microvascular reactivity

The protocol used for assessing vascular relaxation in mice mesenteric microvessels is summarized in the following figure:

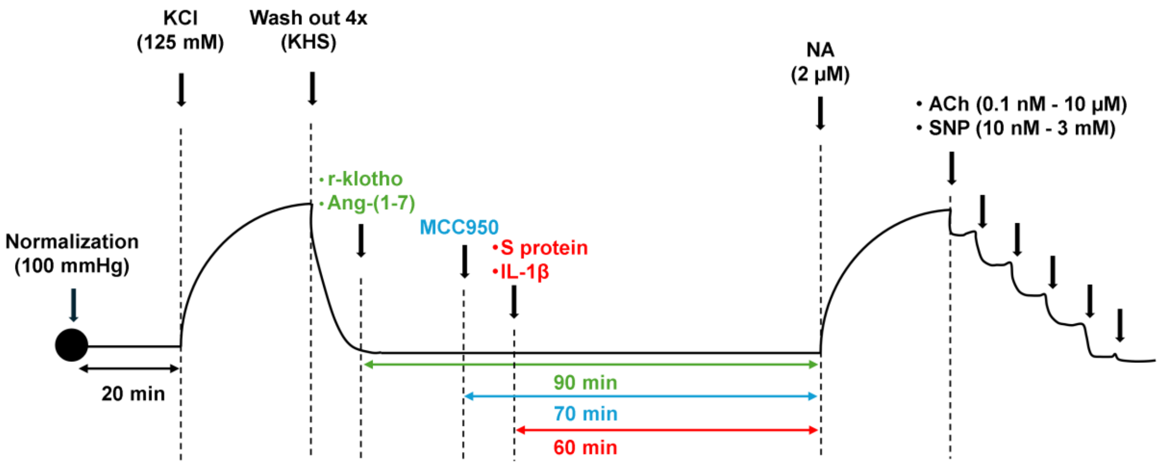

Supplemental Figure 1. Schematic representation of the vascular reactivity experiments protocol. For reactivity experiments, segments from first branch mesenteric arteries were mounted on a small vessel myograph to measure isometric tension. The arterial tensions were normalized to a vascular tone of 100 mmHg for 20 min. Afterwards, they were contracted using 125 mM KCl, followed by 4 washing steps with Krebs-Henseleit solution (KHS). Then, they were treated with protein S (35 nM) or IL-1β (2.5 ng/mL) for 1 h, with or without pretreatment for 30 min with r-klotho (1 nM) or Ang-(1-7) (100 nM). Pretreatment with MCC950 was done for 10 min at 10 µM. After the treatment period, arteries were contracted with noradrenaline (NA) (2 µM) and relaxed with increasing concentrations of acetylcholine (ACh; 0.1 nM to 10 µM) or sodium nitroprusside (SNP; 10 nM to 3 mM).

© 2024. Villacampa A. et al. Published online at http://www.aginganddisease.org/EN/10.14336/AD.2024.0405

SUPPLEMENTARY DATA

Supplemental Tables

Supplemental Table 1. Primers sequences used for the analysis of mRNA levels of heme-oxygenase-1 (HO-1) and klotho.

Primer	Sequence Forward (5'-3')	Sequence Reverse (5'-3')
HO-1	TCCGATGGGTCCTTACACTC	ATTGCCTGGATGTGCTTTTC
Klotho	CAAAGTCTTCGGCCTTGTTC	CTCCCCAAGCAAAGTCACA
18S	GGAAGGGCACCACCAGGAGT	TGCAGCCCCGGACATCTAA

Supplemental Table 2. Values of contraction force induced by noradrenaline (NA) prior to relaxation, pEC_{50}, and % of maximum relaxation in response to acetylcholine (ACh) from the different *ex vivo* treatment groups of Figure 6A.

Ex vivo treatment	Number of segments (number of mice)	NA (2µM)-induced contraction (mN)	pEC_{50} (-logM)	%Maximum relaxation
Control	7 (5)	8.7±0.8	7.6±0.1	77.7±4.1
S (35 nM)	7 (5)	7.1±0.5	6.4±0.2*	64.0±9.5
IL-1β (2.5 ng/mL)	5 (5)	9.1±1.0	5.5±0.3*	54.2±10.9

*p<0.05 versus control

Supplemental Table 3. Values of contraction force induced by noradrenaline (NA) prior to relaxation, pEC_{50}, and % of maximum relaxation, in response to sodium nitroprusside (SNP), from the different *ex vivo* treatment groups of Figure 6B.

Ex vivo treatment	Number of segments (number of mice)	NA (2 µM)-induced contraction (mN)	pEC_{50} (-log M)	%Maximum relaxation
Control	5 (3)	7.8±0.9	4.3±0.2	83.9±8.5
S (35 nM)	3 (3)	7.1±1.0	3.5±0.1	77.4±3.6
IL-1β (2.5 ng/mL)	3 (3)	7.5±0.4	4.0±0.4	74.6±11.4

*p<0.05 versus control

Supplemental Table 4. Values of contraction force induced by noradrenaline (NA) prior to relaxation, pEC_{50}, and % of maximum relaxation in response to acetylcholine (ACh) from the different *ex vivo* treatment groups of Figure 6C.

© 2024. Villacampa A. et al. Published online at http://www.aginganddisease.org/EN/10.14336/AD.2024.0405

SUPPLEMENTARY DATA

Ex vivo treatment	Number of segments (number of mice)	NA (2 μM)-induced contraction (mN)	pEC_{50} (-log M)	%Maximum relaxation
Control	19 (8)	6.4±0.5	7.7±0.08	81.9±3.4
S (35 nM)	13 (8)	6.4±0.4	6.9±0.1*	71.8±6.5
S (35 nM) + MCC950 (10 μM)	4 (4)	7.8±0.7	7.7±0.1#	88.4±5.4

*p<0.05 versus control, #p<0.05 versus S protein (35 nM).

Supplemental Table 5. Values of contraction force induced by noradrenaline (NA) prior to relaxation, pEC_{50}, and % of maximum relaxation in response to acetylcholine (ACh) from the different *ex vivo* treatment groups of Figure 6D and 6E.

Ex vivo treatment	Number of segments (number of mice)	NA (2μM)-induced contraction (mN)	pEC_{50} (-logM)	%Maximum relaxation
Control	12 (4)	7.2±0.7	7.4±0.06	86.6±1.8
S (35 nM)	7 (4)	5.9±0.6	6.7±0.07*	76.1±3.3*
S (35 nM) + Ang-(1-7) (100 nM)	3 (3)	6.7±0.9	7.7±0.09#	95.1±3.2#
S (35 nM) + r-klotho (1 nM)	4 (4)	6.5±0.9	8.1±0.14*#	87.5±68

*p<0.05 versus control, #p<0.05 versus S protein (35 nM).

© 2024. Villacampa A. et al. Published online at http://www.aginganddisease.org/EN/10.14336/AD.2024.0405

SUPPLEMENTARY DATA

Supplemental Figures

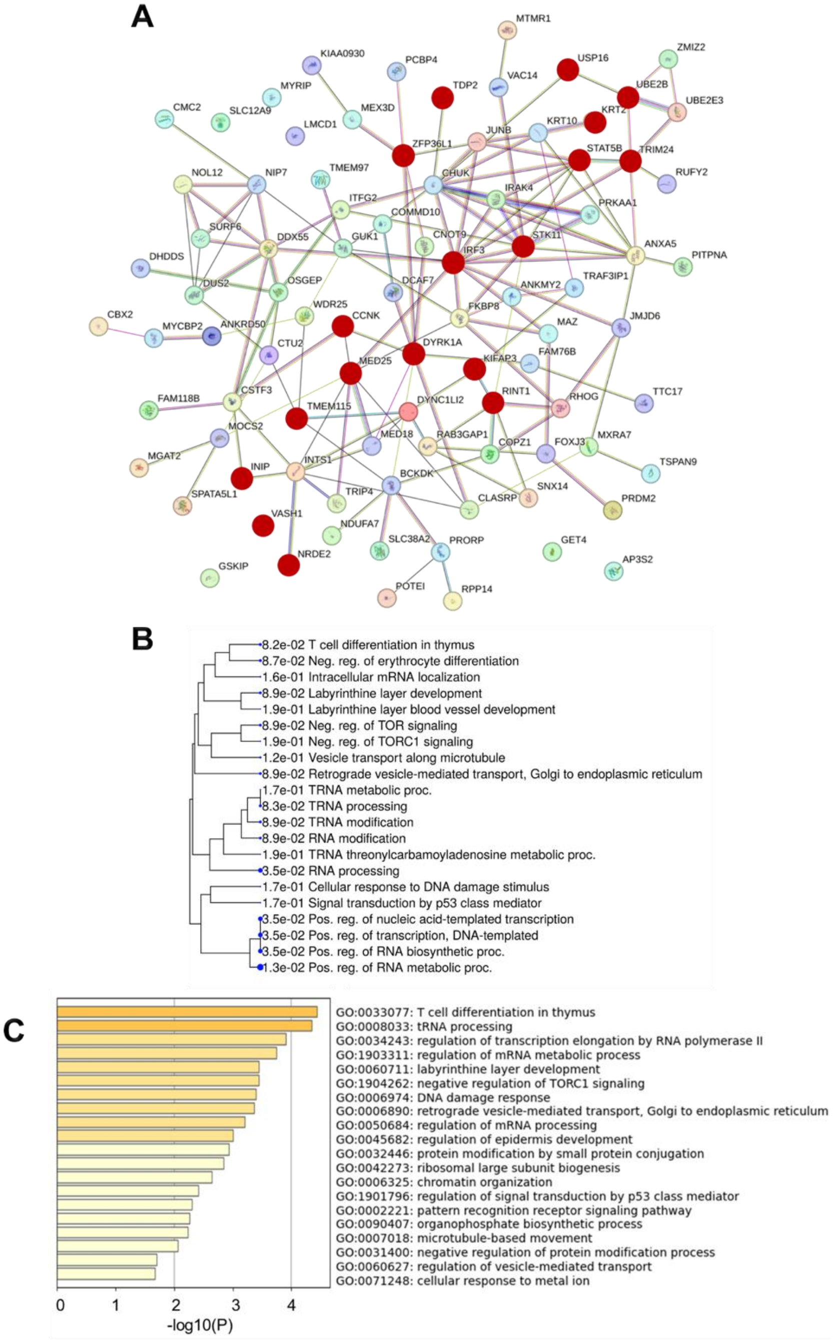

Supplemental Figure 2. S protein changes proteins related to DNA damage response and regulation of signal transduction by p53 class mediator. Human umbilical vein endothelial cells (HUVEC) from 4 different umbilical cords were treated for 18 h with S protein (S; 35 nM) or kept untreated (control). A) 89 differentially expressed proteins between S treatment and control represented with String v12. Proteins in "DNA damage response" (GO:0006974) and "regulation of signal transduction by p53 class mediator" (GO:19017196) categories are highlighted in red. B) Enrichment of GO biological processes of the differentially expressed proteins using ShinyGO. C) Enrichment of GO biological processes of the differentially expressed proteins using Metascape.

© 2024. Villacampa A. et al. Published online at http://www.aginganddisease.org/EN/10.14336/AD.2024.0405

SUPPLEMENTARY DATA

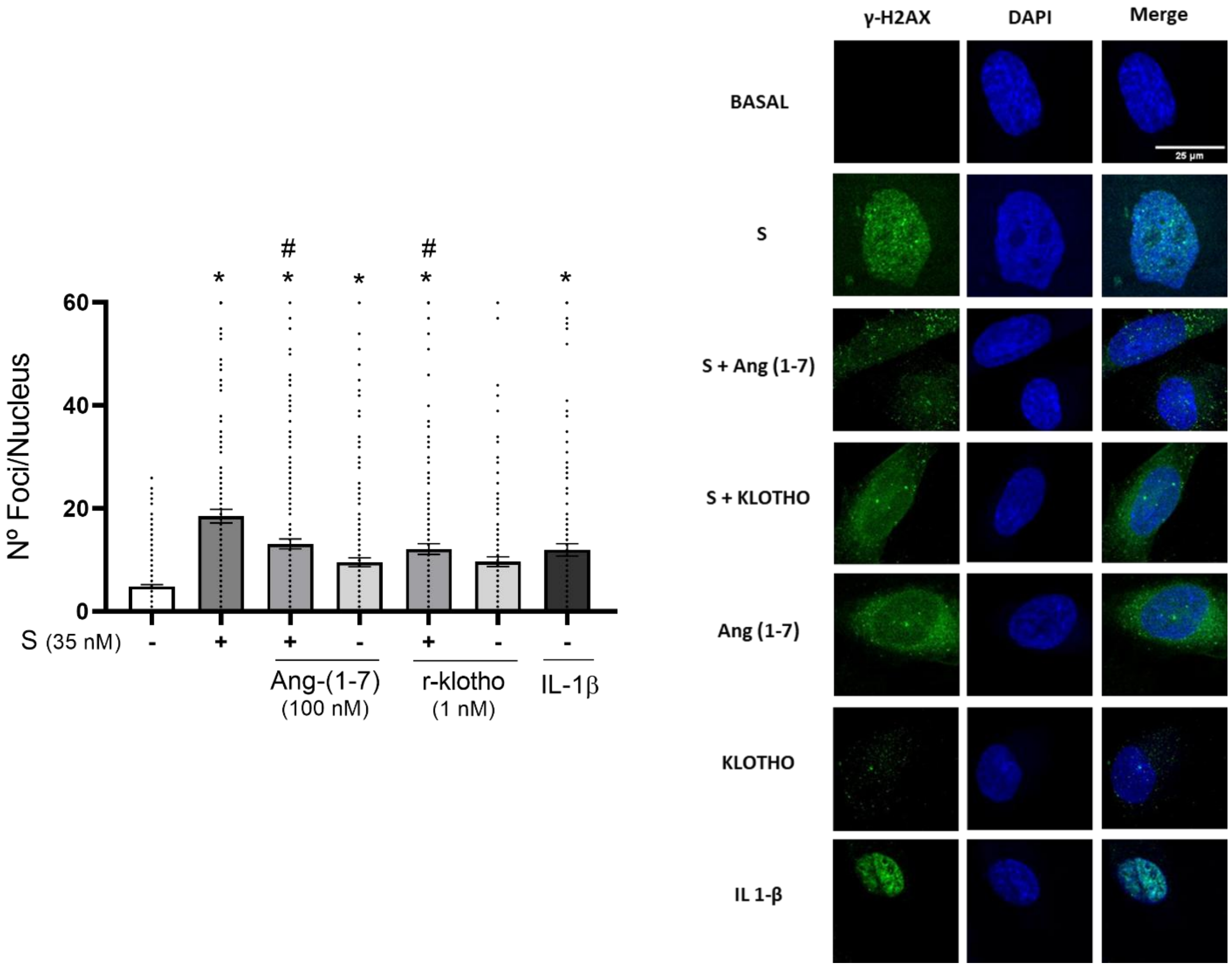

Supplemental Figure 3. S protein increased the number of γH2AX foci and it was prevented with r-klotho and Ang-(1-7). Human umbilical vein endothelial cells (HUVEC) were treated with S protein (35 nM), IL-1β (2.5 ng/mL) and/or Ang-(1-7) (100 nM) or r-klotho (1 nM) for 18 h for the analysis of γH2AX foci by immunofluorescence. Bar graph represents the mean ± SEM. Representative pictures of the immunofluorescence are shown. Statistical differences were tested with Kruskal-Wallis. * p-value < 0.05 vs control. # p-value < 0.05 vs S (35 nM).

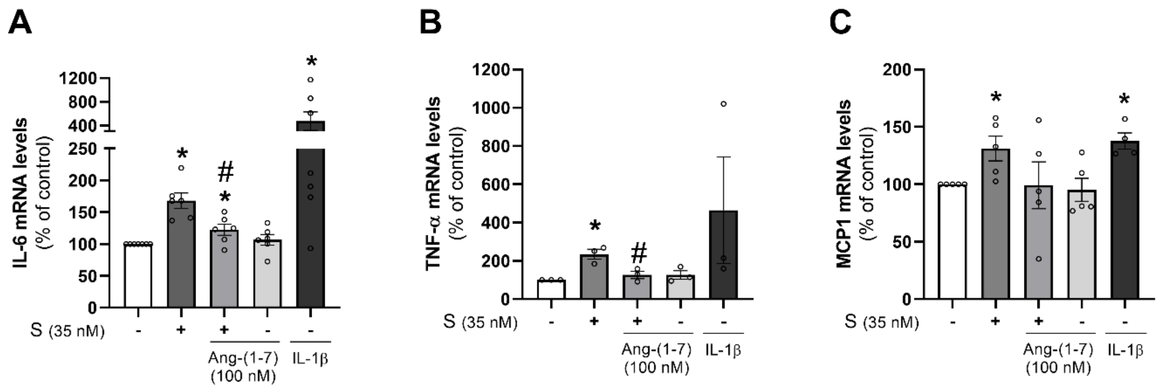

© 2024. Villacampa A. et al. Published online at http://www.aginganddisease.org/EN/10.14336/AD.2024.0405

SUPPLEMENTARY DATA

Supplemental Figure 4. S protein promotes the expression of markers of the senescence-associated secretory phenotype (SASP). Human umbilical vein endothelial cells (HUVEC) were treated for 18 h with IL-1β (2.5 ng/mL) or S protein (S; 35 nM) with or without Ang-(1-7) (100 nM), after which **(A)** IL-6 (n=7, except n=6 for S +Ang-(1-7) and Ang-(1-7) alone), **(B)** TNF-α (n=3) and **(C)** MCP1 (n=5, except n=4 for IL-1β) mRNA levels were determined by RT-qPCR. All bar graphs represent the mean ± SEM. Statistical differences were tested with t-test. * $p < 0.05$ vs control. # $p < 0.05$ vs S (35 nM).

© 2024. Villacampa A. et al. Published online at http://www.aginganddisease.org/EN/10.14336/AD.2024.0405
